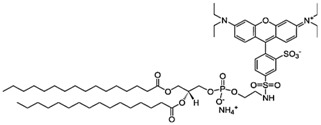# Resveratrol and Grape Extract-loaded Solid Lipid Nanoparticles for the Treatment of Alzheimer’s Disease

**DOI:** 10.3390/molecules22020277

**Published:** 2017-02-13

**Authors:** Joana A. Loureiro, Stephanie Andrade, Ana Duarte, Ana Rute Neves, Joana Fontes Queiroz, Cláudia Nunes, Emmanuel Sevin, Laurence Fenart, Fabien Gosselet, Manuel A. N. Coelho, Maria Carmo Pereira

**Affiliations:** 1 LEPABE, Department of Chemical Engineering, Faculty of Engineering of the University of Porto, Porto 4500-465, Portugal; joana.loureiro@fe.up.pt (J.A.L.); up201306970@fe.up.pt (S.A.); bio12101@fe.up.pt (A.D.); mcoelho@fe.up.pt (M.A.N.C.); 2 UCIBIO, REQUIMTE, Department of Chemical Sciences, Faculty of Pharmacy of the University of Porto, Porto 4050-313, Portugal; rutepneves@gmail.com (A.R.N.); fontes_joana@hotmail.com (J.F.Q.); cdnunes@ff.up.pt (C.N.); 3 Laboratoire de la barrière hémato-encéphalique (LBHE), University Artois, EA 2465, Lens F-62300, France; emmanuel.sevin@univ-artois.fr (E.S.); laurence.tilloy@univ-artois.fr (L.F.); fabien.gosselet@univ-artois.fr (F.G.)

**Keywords:** resveratrol, grape extracts, Alzheimer’s disease, immuno SLN, blood-brain barrier, antibodies, drug delivery systems

## Abstract

The aggregation of amyloid-β peptide (Aβ) has been linked to the formation of neuritic plaques, which are pathological hallmarks of Alzheimer’s disease (AD). Various natural compounds have been suggested as therapeutics for AD. Among these compounds, resveratrol has aroused great interest due to its neuroprotective characteristics. Here, we provide evidence that grape skin and grape seed extracts increase the inhibition effect on Aβ aggregation. However, after intravenous injection, resveratrol is rapidly metabolized into both glucuronic acid and sulfate conjugations of the phenolic groups in the liver and intestinal epithelial cells (within less than 2 h), which are then eliminated. In the present study, we show that solid lipid nanoparticles (SLNs) functionalized with an antibody, the anti-transferrin receptor monoclonal antibody (OX26 mAb), can work as a possible carrier to transport the extract to target the brain. Experiments on human brain-like endothelial cells show that the cellular uptake of the OX26 SLNs is substantially more efficient than that of normal SLNs and SLNs functionalized with an unspecific antibody. As a consequence, the transcytosis ability of these different SLNs is higher when functionalized with OX-26.

## 1. Introduction

Alzheimer’s disease (AD) is classified as the most common form of dementia, accounting more than 80% of dementia cases worldwide [1]. Evidences support a direct association between the degree of dementia of patients that have AD and the concentration of soluble aggregates of Aβ peptide [2,3,4]. The aggregation process of Aβ leads to the formation of insoluble fibrils that have a beta-sheet structure, which accumulate as senile plaque deposits that characterize AD [5,6]. The more abundant sequences of the peptide have 40 (~90%), Aβ_(1–40)_, and 42 (~10%), Aβ_(1–42)_, amino acids [7]. Aβ_(1–42)_ is more toxic and it is the major component of the neuritic plaques found in AD [8,9]. One possible approach to prevent this process could be the reconversion of the Aβ conformation [10,11,12]. Such methodology can be promoted by the addition of a compound capable to remove the beta conformation out of the equilibrium [13,14]. 

Resveratrol (3,5,4′-trihydroxystilbene) is a natural polyphenolic flavonoid, which can be found in nature as both *cis* and *trans* isomers, the latter considered to be the most abundant and biologically active [15]. Several effects have been related with the intake of resveratrol, such as anti-carcinogenic, anti-inflammatory, anti-obesity and heart/brain protective effects [15]. The neuroprotective effects of resveratrol in neurological diseases are related to the protection of neurons against oxidative damage and toxicity, and to the prevention of apoptotic neuronal death [15,16]. Resveratrol can be found in the seeds and skins of grapes, red wine, mulberries, peanuts, rhubarb and in several other plants [16]. Its concentration in the skin and seeds of grapes is approximately 50–100 µg per gram, corresponding to 5%–10% of their biomass; however, it varies considerably on different grape cultivation methods [15,16]. This fact is associated with the *French Paradox*, which refers to the beneficial effects of a moderate consumption of red wine. Besides resveratrol, both red wine and purple grapes (specifically the skin and seeds) contain several flavonoids, such as quercetins, catechins, gallocatechins, procyanidins, and prodelphidins [17].

After intravenous injection, resveratrol is rapidly (within less than 2 h) metabolized in the liver and intestinal epithelial cells into both glucuronic acid and sulfate phenolic group conjugates which are then eliminated [17]. Thus, resveratrol has low bioavailability limiting its biological and pharmacological benefits. It also has poor water solubility and is chemically unstable, being degraded by isomerization when exposed to elevated temperatures, pH changes, UV light or certain types of enzymes [15]. Moreover, to reach the brain where resveratrol may demonstrate its neuroprotective effects it has to cross the blood-brain barrier (BBB), a specialized barrier composed of brain endothelial cells which strictly control the exchanges of molecules between the brain and the blood. Thus, this warrants a need to develop an efficient system to increase the resveratrol bioavailability and to improve its distribution into the brain.

Solid lipid nanoparticles (SLNs) are stable lipid-based nanoparticles composed by a solid hydrophobic lipid core where the therapeutic drug can be dissolved or dispersed. These nanoparticles are made of an oil/water emulsion with lipids that are solid at room temperature and body temperature [18,19,20]. Their size is between 40 to 200 nm providing them the ability to bypass liver and spleen filtration, escape from the reticulo-endothelial system (RES), and thus cross the tight endothelial cells of the BBB [21,22,23,24].

Following intravenous injection, nanoparticles are rapidly opsonized and cleared from the blood stream by the macrophages of the RES which are mainly localized in the liver and spleen [25,26]. However, the blood circulation time of the nanoparticle can be prolonged by modification of the nanoparticle surface with surfactants such as polysorbates or by covalent attachment of hydrophilic polyethylene glycol (PEG) chains to the core polymer [27]. Besides increasing the blood circulation time, PEG chains grant steric stabilization of the nanoparticle surface allowing the attachment of ligands (e.g., antibodies, proteins or aptamers) capable of binding to BBB nutrient transport systems or internalizing receptors [28,29].

The selection of the ligands is extremely critical since the receptor should be preferentially expressed at the BBB, but ideally it should be brain specific in order to reduce potential side-effects and increase transport efficiency [30,31,32]. Also, the natural saturation of the receptor must be considered to avoid competition with the natural ligand [28,32]. The specific monoclonal antibody (mAb) type OX26 has been well described to bind cells that express the transferrin receptors (TfR), such as the BBB cells [33,34,35].

This study aimed to develop a targeted therapeutic system for intravenous administration of resveratrol and grape skin and seed extracts using SLNs. The lipid nanoparticles were also functionalized with an OX26 mAb. The nanoparticles were characterized morphologically by size, zeta potential and entrapment efficiency. The ability of these SLNs to cross the BBB was assessed using an in vitro model of the human BBB [36]. These results demonstrate that the nanoparticles synthetized are able to encapsulate the extracts and can be functionalized to cross the BBB. These attributes make them promising tool to aid in therapy for the treatment of AD.

## 2. Results and Discussion

### 2.1. Impact of Resveratrol and Extracts of Grape Skin and Seed on Aβ_(1–42)_ Fibrillation

**Figure 1:**
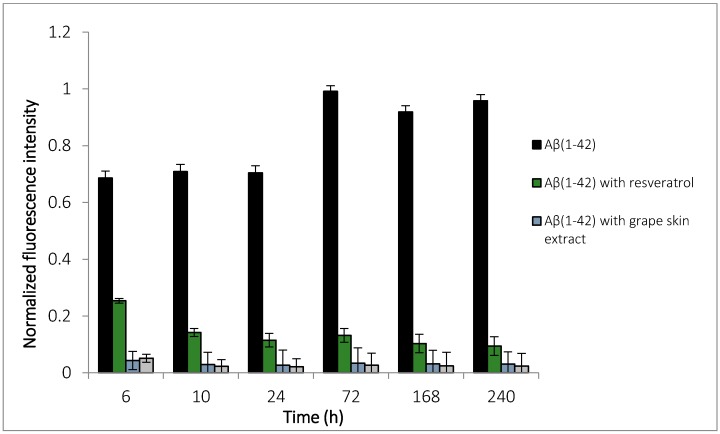
Effect of the resveratrol and extracts of the grape skin and grape seed on Aβ_(1–42)_ fibrils content as monitored by Thioflavin T fluorescence. The Aβ_(1–42)_ concentration was 25 μM, and the resveratrol and extracts concentration was 40 μM. The samples were incubated at 37 °C for 10 days in PBS buffer.

The aggregation of Aβ_(1–42)_ incubated at 37 °C with resveratrol and grape extracts (skin and seed) at a molar ratio of 5:8 and 5:16 (Aβ_(1–42)_:resveratrol or extracts) was evaluated by the ThT binding assay (Figure 1). In solution ThT is a poor fluorophore, the excitation energy dissipates through rotation around the central axis in its molecular structure. In the presence of β-sheet fibrils, ThT can enter into cavities produced by the quaternary structure of the protein binding with the protein. The rotation becomes restricted and results in an increase in fluorescence quantum yield [37,38]. The intensity of fluorescence is proportional to the quantity of fibril formation (450 nm) [39,40]. At 6 h incubation time at a molar ratio of 5:8, the ThT fluorescence intensity for the sample with Aβ_(1–42_) alone presents a significant high fluorescence suggesting the presence of amyloid fibrils. However, for the samples of Aβ_(1–42)_ that contain the grape extracts or resveratrol, the fluorescence is low, indicating a lower content of amyloid fibrils. All samples reached a stable signal after approximately 10 h incubation. The fluorescence of ThT in the presence of Aβ_(1–42)_ and extracts or resveratrol is very slow, demonstrating that the grape skin extract inhibits around 92% and the grape seed extract inhibits around 97%. Resveratrol inhibits around 86% of the Aβ_(1–42)_ peptide aggregation. This inhibition of fibrillation is more relevant for the extract when compared with the pure resveratrol. The extracts and resveratrol in the absence of Aβ_(1–42_) do not induce any change in the fluorescence signal of ThT.

At the concentration of 80 μM, these values increased for 91%, 97% and 98%, respectively (data not shown). No significant changes of percentage of Aβ_(1–42)_ aggregation was observed for both concentrations used. 

The results suggest that beyond to contain resveratrol that inhibits the aggregation of Aβ_(1–42)_, the extracts contain other polyphenols that make this inhibitory effect most pronounced.

**Figure 2:**
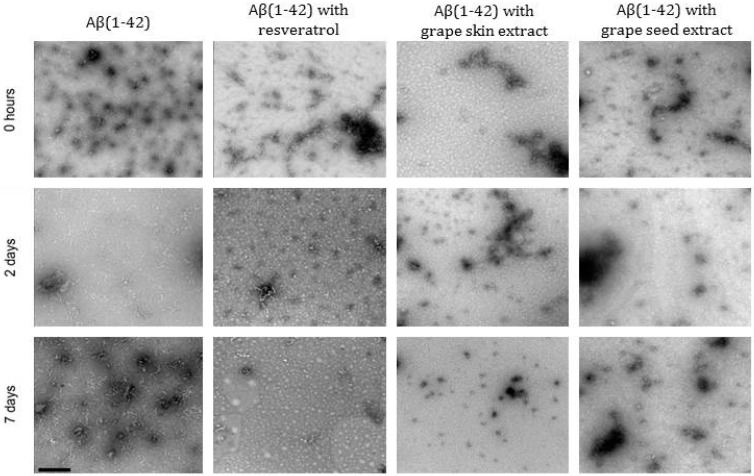
Transmission electron microscopy analysis of the effect of the resveratrol and extracts of the grape skin and grape seed on Aβ_(1–42)_ aggregation. The Aβ_(1–42)_ concentration was 25 μΜ and the resveratrol and extracts concentration was 80 μM. The samples were incubated at 37 °C in phosphate buffered saline buffer. The scale bar corresponds to 100 nm.

TEM analysis of samples confirmed the inhibition of Aβ_(1–42)_ aggregation both in the presence of resveratrol and extracts (Figure 2). The Aβ_(1–42)_ alone showed fibril formation whereas in the presence of resveratrol or extracts, the samples appeared to be devoid of those structures. When Aβ_(1–42)_ was co-incubated with resveratrol and extracts, the number of fibrils decreased. There were some oligomers present in the samples but only small aggregates were observed indicating the fibrillogenesis inhibition.

In support of these results, Hung, et al. studied the Aβ_(1–40)_ aggregation in the presence and absence of resveratrol using ThT and TEM. In the presence of resveratrol (50 μM), Aβ fibril formation was greatly attenuated [41]. Also, Marambaud, et al. demonstrated that resveratrol causes a decrease in Aβ levels. To this end, they treated APP695-transfected HEK293 cells with increasing concentrations of resveratrol and analyzed Aβ levels by ELISA and western blot (WB). Total secreted Aβ, including Aβ_(1–40)_ and Aβ_(1–42)_, was markedly reduced by 20–40 μM resveratrol after 24 h of incubation [42]. Therefore, resveratrol could be efficient to treat AD by 2 potential processes—inhibition of fibril formation and decrease of amyloid synthesis.

### 2.2. Solid Lipid Nanoparticles Formulation

To circumvent the resveratrol limitations related with its bioavailability, SLN nanoparticles were produced as a carrier. The method used for the nanoparticle synthesis is a compromise between the high shear homogenization method and the ultrasonication method. Hence, it is possible to produce particles with a micrometer size by the first method, and then, reduce their size to the nanometer size range by the second method [19,43].

The mean size of the unloaded nanoparticles measured by dynamic light scattering (DLS) was 142 ± 10 nm. The PDI of the unloaded SLN was 0.12 ± 0.04 showing that the formulation had a monodisperse population. The zeta potential of the nanoparticles was −0.08 ± 0.05 mV. The process yield of the nanoparticles filtered through a 200 nm filter was 48% ± 6%.

Formulations with different concentrations of grape seed and skin extracts, 2, 5, 10 and 15 mg were prepared. The grape extract loaded-nanoparticles mean sizes are presented in Table 1. All formulations showed a homogeneous size distribution with a mean diameter between 168 and 189 nm which corresponds to the required size for brain drug delivery [44,45].

For the unloaded SLN, the zeta potential of the different extract-loaded nanoparticles was almost neutral, between −0.34 and 0.35 mV, meaning that the encapsulation of these compounds did not have a significant impact on the zeta potential suggesting that the extracts were encapsulated inside of the SLN. This is confirmed by the fact that resveratrol has a lipophilic nature, thus its preferential localization should be in the nanoparticle’s core. The same happened with the grape extracts, even though they are constituted by hydrophilic and lipophilic compounds, the extracts were sucessfully encapsulated in the SLNs.

The encapsulation efficiency of each formulation is shown in Table 2. The percentage of encapsulation was high, between 75% and 100%, suggesting that the solid lipid nanoparticles are a suitable system for the incorporation of both grape extracts and resveratrol. It is also possible to observe that upon increasing the extract concentration, the entrapment efficiency decreases smoothly as it would be expected due to the SLNs’ saturation. 

Taking the previous results in consideration, the final extract concentration used in the following experiments was 10 mg. The same analysis was done for resveratrol loaded SLN (10 mg resveratrol encapsulated) and nanoparticles with 176 ± 24 nm of diameter, PI 0.16 ± 0.10 and 0.17 mV of zeta potential were obtained. The EE was 94% ± 9%.

**Figure 3:**
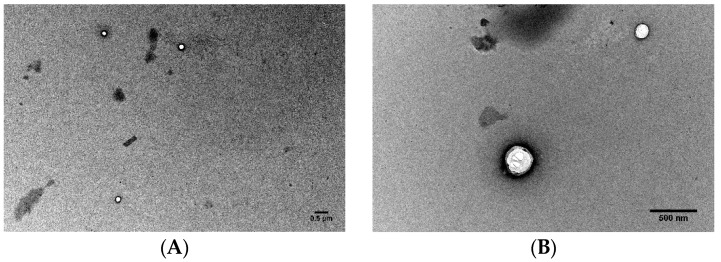

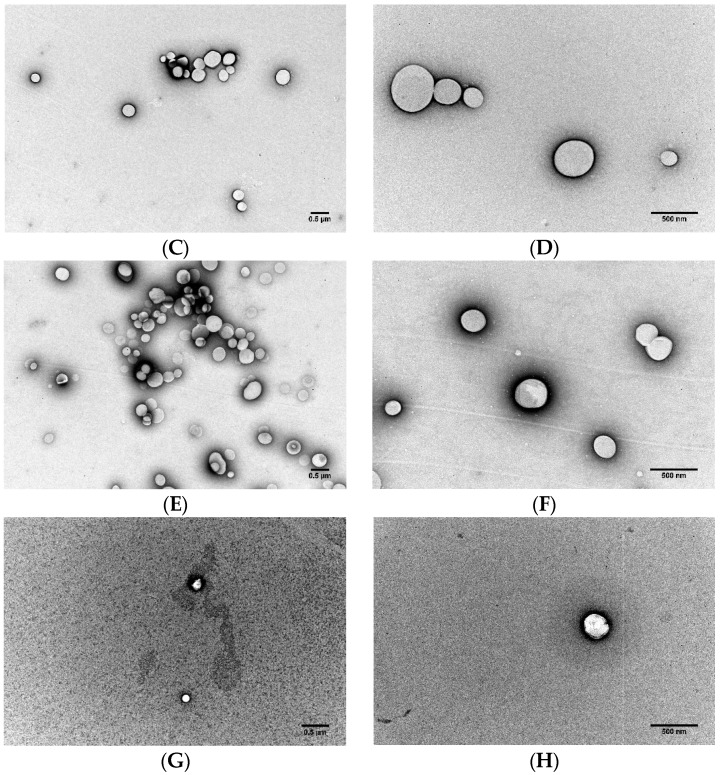
Transmission electron microscopy images of unloaded SLN (**A**,**B**), SLN with resveratrol encapsulated (**C**,**D**), SLN with skin grape extract encapsulated (**E**,**F**) and SLN with seeds grape extract encapsulated (**G**,**H**). Samples were diluted at a ratio of 1:100. Scale bar: 500 nm.

The morphology of the lipid nanoparticles was observed by TEM (Figure 3). The images revealed that the nanoparticles were spherical and with a uniform shape with smooth surfaces. Also, TEM analysis confirmed the sizes previously measured by DLS. Moreover, it is possible to observe that the nanoparticles’ shape did not seem to be altered when loaded with grape extracts and resveratrol. 

### 2.3. Solid Lipid Nanoparticle Stability

To study the stability of unloaded SLNs and with resveratrol and extracts encapsulated, the size and zeta potential of carriers were evaluated during 2 months at RT. Variation of the size and zeta potential means that the structures of nanoparticles rearrange, i.e., the structures will be different from the initial ones, and the nanoparticles lose their stability [46,47]. Over time, there were no significant variations on the size which indicated that the SLN were stable during this period (Table 3). Also the zeta potential values are not significantly different from each other, maintaining around 0 mV. Thus, this assures that the SLNs steric stability has not been affected during this period.

One parameter to access the stability of the different formulations is the entrapment efficiency. It is known that SLNs have a highly organized matrix with a tendency to form perfect crystals over time which can eventually lead to an expulsion of the drug during storage. However, the results of entrapment efficiency shown in Table 4 demonstrated that the nanoparticles were able to retain the initial amount of encapsulated grape extracts during a 2 month assessment.

### 2.4. Effect of the Loaded Solid Lipid Nanoparticles on Amyloid-β Aggregation

**Figure 4:**
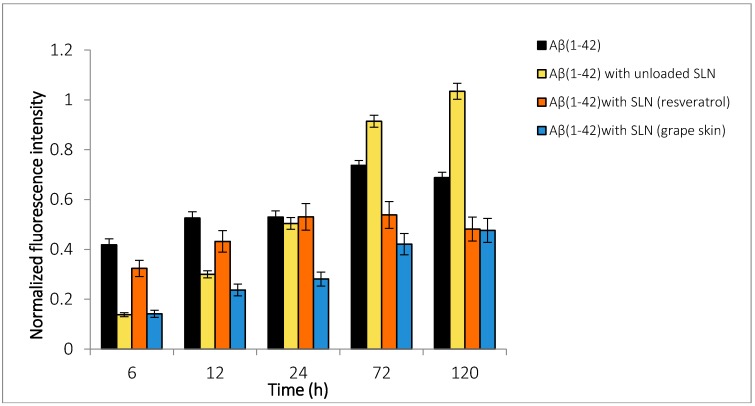
Effect of the interaction of the loaded-nanoparticles (resveratrol and grape skin extract) and unloaded-nanoparticles on Aβ_(1–42)_ fibrils content as monitored by Thioflavin T fluorescence. The Aβ_(1–42)_ concentration was 25 μM, and the resveratrol and extract concentration was 40 μM. The samples were incubated at 37 °C for 10 days in the presence or absence of resveratrol and extract of the grape skin in phosphate buffered saline buffer.

To evaluate how the SLNs encapsulated with different compounds interact with the Aβ_(1–42)_ and reduce/induce the aggregation of these peptides, a kinetics studies were performed. A decrease in the peptide aggregation was observed when resveratrol and the grape extracts are encapsulated (Figure 4). After 3 days incubation (72 h) it is observed that the resveratrol encapsulated in the SLN reduces 26% of Aβ_(1–42)_, and the encapsulated extracts reduced by 31%. On the other hand, results also showed that the unloaded nanoparticles acted as nucleus that promotes the aggregation of the Aβ_(1–42)_. Here, we observed that the SLNs release the beneficial compounds in a controlled way preventing Aβ peptide fibrillation.

### 2.5. Conjugation of the Antibodies

To make these particles more selective, OX26 mAb, which recognize the TfR that are overexpressed in the BBB, were conjugated. To compare the ability of the OX26 to reach the brain SLNs were also conjugated with the mAb LB 509 that recognizes α-synuclein, a protein that is non-specific to the BBB TfR. As a control, nanoparticles without conjugated antibodies were synthetized. Covalent coupling methods for attaching the antibodies at the PEG terminus were based on functionalized PEG with a chemically reactive end-group, the PEG-maleimide (thiol reactive). SLN size was determined after each step of preparation (Table 4) to confirm an efficient conjugation. A small, but significant increase of the hydrodynamic diameters was observed due to the binding of mAbs to the surface of SLNs. The size of the SLNs increased 16 to 22 nm after the addition of LB 509 mAb or OX26 respectively, which corresponds approximately to the diameter of globular mAbs [48]. The addition of the mAb to the SLN does not significantly affect the zeta potential of the nanocarriers.

In order to study the stability of SLN conjugated with the mAb, the size and the zeta potential of carriers were evaluated during 1 month. Over this time, there were no significant variations on these parameters which indicate that the immuno SLN were stable for this period (data not shown). 

The binding of the mAb to the nanoparticles was also determined by ELISA. Significantly higher absorbance at 405 nm was observed in the nanoparticles conjugated with the two different types of mAbs (1.6 ± 0.3) when compared with the nanoparticles without mAb (0.07 ± 0.02). Therefore, the antibody used demonstrated bioactivity for the transferrin receptor. 

### 2.6. Uptake and Transport Assays

**Figure 5:**
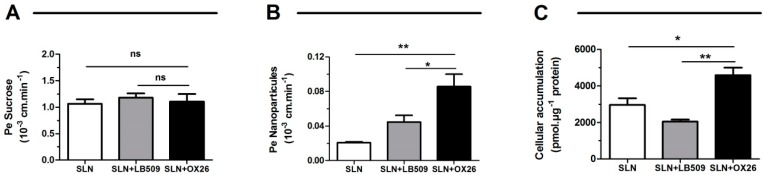
Permeability and cellular accumulation of 250 µM of SLN particles coupled with and without antibodies in an in vitro model of the human blood-brain barrier. SLN particles were incubated in the luminal compartment for 2 h. At 30, 60 and 120 min, media was aliquoted and sucrose permeability (**A**), SLN transport (**B**) and SLN cellular accumulation (**C**) were measured. Results represent the means ± SEM (*n* = 6). ns: non-significant, * <0.05, ** <0.01.

Before testing the uptake and transport of the different SLN functionalized or not with antibodies, their potential toxicity was first assessed in an in vitro model of the human BBB expressing TfR [49]. Different concentrations (0 to 250 µM) of the different SLNs were added in the luminal compartment (representing the blood), and the endothelial permeability (Pe) to sucrose (a BBB integrity marker) was measured. No significant changes in Pe values for the SLN concentrations tested here (0–250 µM) was observed (data not shown). Hence, a concentration of 250 µM was used in subsequent experiments (Figure 5A). 

As shown in Figure 5B, the transport of the SLN functionalized with OX26 across the HBLEC monolayer is almost 2-fold higher than the SLN functionalized with LB 509 and 4-fold higher than the SLN alone (Pe = (0.086 ± 0.014) × 10^−3^ cm/min versus Pe = (0.045 ± 0.008) × 10^−3^ cm/min and Pe = (0.021 ± 0.001) × 10^−3^ cm/min, respectively). In addition, the SLN functionalized with OX26 showed a significant higher intracellular accumulation when compared with SLN functionalized with LB 509 and SLN alone (4588 ± 410 pmol/µg of proteins versus 2038 ± 110 pmol/µg of proteins and 2951 ± 373 pmol/µg of proteins, respectively) (Figure 5C). In future, in vivo studies will be performed to evaluate the actual potential of these nanocarriers.

## 3. Materials and Methods

### 3.1. Stock Solutions of Amyloid-β Peptide

Aβ_(1–42)_ (amyloid-β peptide 1-42, purity >95.22%, MW: 4514.14, Selleck Chemicals, Houston, TX, USA) was dissolved in 1,1,1,3,3,3-hexafluoro-2-propanol (HFIP, >99.8%, Sigma-Aldrich, St. Louis, MO, USA) at a concentration of 1.0 mg/mL. HFIP was evaporated with nitrogen flow, and the peptide film was dissolved in DMSO (dimethyl sulfoxide for molecular biology, >99.9%, FW: 78.13, Sigma-Aldrich) at a concentration of 9.0 mg/mL.

### 3.2. Stock Solutions of Resveratrol, Extracts of Grape Seed and Skin

Resveratrol (3,5,4′-trihydroxystilbene, ≥99%, MW 228.24, Sigma-Aldrich) and the extracts of the grape seed and grape skin (purity ≥95%, Monteloeder, Alicante, Spain) were dissolved in 10 mM phosphate buffered saline (PBS), (pH 7.4, 2.7 mM potassium chloride and 137 mM sodium chloride, Sigma-Aldrich) at a concentration of 80 μM. To ensure complete dissolution the solutions were placed in a water bath at 70 °C for 10 min. 

### 3.3. Thioflavin T Binding Assay

Interaction of the resveratrol and extracts of grape skin and grape seed (40 and 80 µM) with Aβ_(1–42)_ (25 µM) was evaluated through the Thioflavin T (ThT) binding assay. The samples were incubated at 37 °C for 10 days. A ThT stock solution was prepared in PBS at the concentration of 0.8 mg/mL, and a ThT working solution was prepared by diluting 1 mL of the stock solution in 50 mL of PBS buffer. The ThT solution was filtered using a 0.2 nm syringe. The fibrils conjugated with ThT have the excitation maximum at 450 nm and enhanced emission at 482 nm [50]. The fluorescence intensity was measured using a Synergy 2 fluorescence spectrometer (BioTek, Winooski, VT, USA) with the excitation filter set at 420/50 nm and the emission filter at 485/20 nm.

### 3.4. Transmission Electron Microscopy

Aβ_(1–42)_ (25 µM) was incubated at 37 °C with the resveratrol and with the extracts (80 µM) in PBS buffer for 7 days. Five microliters of each sample were placed on carbon-formvar coated 400 mesh spacing grids and left to adsorb for 5 min. Negative staining was performed with 2% filtered aqueous solution of uranyl acetate for 45 s. The grids were visualized using a JEM 1400 electron microscope (Jeol, Tokyo, Japan) at 80 kV.

### 3.5. Solid Lipid Nanoparticles Preparation

The lipid phase, containing the solid lipid cetylpalmitate (Gattefossé, Lyon, France) (500 mg) and the stabilizer polysorbate 80 (Tween^®^ 80 Sigma-Aldrich) (150 mg) was melted at 70 °C, which is above the lipid’s melting point (Table 5). The melted lipid was then dispersed in ultrapure water (4.35 mL) (Milli-Q RG) at the same temperature by high-speed stirring in an Ultra-Turrax T25 (Janke and Kunkel IKA-Labortechnik, Staufen, Germany) followed by sonication (70% amplitude) using a Vibra-Cell™ CV18 (15 min, Sonics and Materials, Newtown, CT, USA). The nanoemulsion was left to cool at room temperature to allow the crystallization of the lipid and consequent formation of the solid lipid nanoparticles [19,44].

The final parameters chosen were a high shear homogenization of 2 min at 12,000 rpm and a 15 min sonication at an intensity of 70% (the parameters of both techniques were previously optimized to establish the best conditions for the production of a stable formulation with an average size of less than 200 nm) [19,43]. 

For the nanoparticles conjugated with mAb, 0.5 mg of DSPE-PEG_2000_-maleimide (1,2-distearoyl-*sn*-glycero-3-phosphoethanolamine-*N*-[maleimide(polyethylene glycol)-2000] ammonium salt, Avanti Polar Lipids, Alabaster, AL, USA) and 0.5 mg of LissRhod-PE (1,2-dipalmitoyl-*sn*-glycero-3-phosphoethanolamine-*N*-(lissamine rhodamine B sulfonyl) ammonium salt, Avanti Polar Lipids) was added to the lipid phase and then melted at 70 °C.

The particle size and surface charge (zeta potential) of the NPs suspensions were analyzed using a Zetasizer Nano ZS (Malvern Instruments Ltd., Malvern, UK). The SLNs size measurements were based upon photon correlation spectroscopy. With this technique the hydrodynamic diameter and the polydispersity index (PDI), which is a dimensionless measure for the broadness of the particle size distribution, were obtained. The zeta potential was analyzed by laser Doppler velocimetry using the same instrument.

### 3.6. Determination of the Yield

The yield was determined by calculating the weight difference of the samples before and after filtration (Equation (1)):

(1) $$Yield\;\left(\%\right)=\frac{remaining\;lipid\;after\;filtration}{remaining\;lipid\;before\;filtration}\times 100$$

In order to do this, two sets of samples, one filtered and the other non-filtered, were dried and the remaining lipid was weighed.

### 3.7. Extracts Encapsulation and Release

Extracts were encapsulated in the lipid nanoparticles. To encapsulate the extracts, they were added to the lipids mixture and melted at 70 °C. The entrapment efficiency (EE) of the compounds was determined by the difference between the amount used in the formulation synthesis and the amount that remained free in the aqueous phase, as follows:

(2) $$\%EE=1-\frac{Unentrapped\;extract}{Total\;amount\;of\;extract}\times 100$$

Samples of the different formulations were diluted in ultrapure water (1:200), transferred into Amicon^®^ Ultra Centrifugal Filters (Merck Millipore, Billerica, MS, USA), and centrifuged using an Allegra^®^ X-15R Centrifuge (Beckman Coulter, Pasadena, CA, USA) during 25 min at 4300 rpm. Afterwards, the free extracts present in the supernatant were collected and quantified using a V-660 spectrophotometer (Jasco, Easton, MD, USA).

The release of the extracts from the SLNs was studied by using a dialysis bag (Float-A-Lyzer G2, CE, 100 kDa, Spectrum Labs, Houston, TX, USA). A volume of 2 mL of SLNs was added into the dialysis tube, and dialysis was carried out against PBS buffer at 37 °C with continuous stirring (200 rpm). Aliquots from the medium outside the dialysis bag were collected at different times. The percentage of the released extract at each time point was then calculated using Equation (3):

(3) $$\%\;of\;extract\;released=\frac{Extract\;released}{Total\;amount\;of\;extract}\times 100$$

### 3.8. Conjugation of the Antibodies

Covalent coupling methods for attaching the antibodies at the PEG terminus by using functionalized PEG with a chemically reactive end-group maleimide were applied. For the maleimide-mAb conjugation, the mAb (OX26 (AbD Serotec, Oxford, UK) and LB 509 (Abcam, Cambridge, UK) were activated by a 20X molar excess of Traut’s reagent (2-iminothiolane hydrochloride, MW 137.73, Sigma-Aldrich). A drop of EDTA (ethylenediaminetetraacetic acid, MW 292.40, Sigma-Aldrich) 0.28 M was added to prevent metal catalyzed oxidation of the sulfhydryl groups [51]. It is important to note that the thiolation of the mAb does not interfere with their binding site [52]; however, the maleimide group can be hydrolyzed when in contact with water, so it is highly recommended to conjugate the mAb immediately after the nanoparticle synthesis.

The unreacted EDTA/2-iminothiolane complexes were removed by application of size exclusion chromatography using a Sephadex column PD-Minitrap G25 (GE Healthcare, Little Chalfont, UK). After, the antibodies were incubated at room temperature for 1 h and then at 4 °C overnight. The antibodies were added to the SLNs at a molar ratio of 1:1 between antibodies and functionalized PEG.

The affinity of the conjugated SLNs for TfR was analyzed by ELISA. For this, the surface of a 96-well plate (flat-bottom MaxiSorp^®^, Nunc, Waltham, MA, USA) was coated with TfR during 1 h at 37 °C. The plate was then blocked with bovine serum albumin (BSA, ~66 kDa) and incubated for another hour at 37 °C, followed by the addition of the nanoparticles to each well. After incubation and subsequent washing, the secondary antibody, which was conjugated with peroxidase, was react during 45 min at room temperature. The revealing solution was composed by citric acid (MW 210.14, Sigma-Aldrich), ABTS (2,2′-azino-bis(3-ethylbenzothiazoline-6-sulfonic acid) diammonium salt, MW 548.68, Sigma Aldrich) and H_2_O_2_ (hydrogen peroxide solution, MW 34.02, Sigma-Aldrich), and the absorbance spectrum of each well was read at 405 nm using the BioTek Synergy 2 spectrometer. Nanoparticles without being conjugated were used as a negative control.

### 3.9. The In Vitro Model of the Human Blood-Brain Barrier 

The in vitro BBB model was established by culturing endothelial cells (ECs) derived from hematopoietic stem cells isolated from umbilical cord blood on the upper side of a filter insert, and pericytes at the bottom of the well, as previously described [49,53]. All the donors of umbilical cord blood samples had given their written informed consent in compliance with French legislation. The sample collection was approved by the local investigational review board (Béthune Maternity Hospital, Beuvry, France). The study’s objectives and protocol were approved by the French Ministry of Higher Education and Research (reference; CODECOH DC2011-1321). All experiments were carried out in line with the authorized protocol. Briefly, mononuclear cells were isolated from human umbilical cord blood after Ficoll (Histopaque-1077 Hybri Max; Sigma-Aldrich) density gradient separation. Then, CD34^+^ cells were positively selected using the mini-MACS immunomagnetic separation system (Miltenyi Biotec, Bergisch Gladbach, Germany) according to the manufacturer’s recommendations. Afterwards, CD34^+^ cells were differentiated into endothelial cells according to a protocol previously reported by Pedroso and collaborators [54]. Isolated CD34^+^ cells were cultured in Endothelial Cell Medium (ECM; ScienCell, Carlsbad, CA, USA) supplemented with 20% (*v*/*v*) fetal bovine serum (FBS, Invitrogen, Cergy-Pontoise, France) and 50 ng/mL of VEGF165 (PrepoTech Inc., Neuilly-Sur-Seine, France), on 0.2% gelatin-coated 24-well plates (2 × 10^5^ cells/well). After 15–20 days ECs were seeded in the culture dish. For each experiment, the cells were expanded in 0.2% (*w*/*v*) gelatin-coated 100mm-Petri Dishes (Corning) in ECM medium supplemented with 5% (*v*/*v*) FBS, 50 µg/mL gentamycin (Biochrom AG, Berlin, Germany) and 1 ng/mL bFGF, until confluence and then trypsinized and seeded at a density of 8 × 10^4^ onto coated inserts (Transwell, 3-µm-pore inserts). These inserts with cells were maintained with a dry bottom for one week (500 µL of medium in the upper compartment, changed every other day) to avoid the cells crossing the membrane and forming a non-physiological second layer on the lower face of the insert. Next, the inserts were transferred onto pericytes (50,000 cells per well, seeded in 12-well plates two days before the transfer). The resulting co-culture was maintained for 5 days—the minimum length of time required for the induction of barrier properties [49,53,55]—under standard conditions (a humidified 5% CO_2_ atmosphere, with renewal of the ECM-5 medium every two days). Under these conditions, ECs exhibit most of the characteristics of the BBB such as low permeability to non-permeable markers (sucrose, fluorescein sodium) and high TEER [41,42]. For this reason, these cells are then considered to be human brain-like endothelial cells (HBLECs).

### 3.10. Permeability Experiments and Cellular Accumulation

The inserts (in a 12-well format, containing an HBLEC layer or merely coated) were transferred into 12-well plates containing 1.5 mL of Ringer-HEPES solution (150 mM NaCl, 5.2 mM KCl, 2.2 mM CaCl_2_, 0.2 mM MgCl_2_-6H_2_O, 6 mM NaHCO_3_, 5 mM HEPES, 2.8 mM glucose; pH 7.4) per well (constituting the abluminal compartment). The insert’s cell culture medium was removed, and 0.5 mL of Ringer-HEPES solution containing 250 μM SLN nanoparticles (NPs) was added to the upper (luminal) compartment and containing 1.5 KBq/mL of radiolabeled ^14^C-sucrose (Perkin Elmer, Waltham, MA, USA). All incubations were performed at 37 °C under gentle agitation. After 30, 60 and 120 min, aliquots from each lower and upper compartment were taken. Cellular layers were rinsed 5 times with cold Ringer HEPES solution and lysed with 250 µL of RIPA buffer (Sigma Aldrich). Samples were analyzed using Liquid Scintillation Analyser (Tri-carb 2100TR, Perkin Elmer) for ^14^C-sucrose and fluorimeter (Synergy™ H1, BioTek) for NP quantification (excitation wavelength 538 nm; emission wavelength: 586 nm, gain 100, 12 measurements per value). The endothelial permeability coefficient (Pe) of ^14^C-sucrose and NPs was calculated in cm/min as previously described [44]. The clearance principle was used to obtain a concentration-independent index of transport. Briefly, the mean volume cleared is plotted versus time, and the slope is estimated by linear regression. The permeability values of the insert (PSf, for inserts with a coating only) and the insert plus endothelium (PSt, for inserts with a coating and cells) were taken into consideration by applying the following equation: 1/PSe = 1/PSt − 1/PSf. To obtain the endothelial permeability coefficient (Pe, in cm/min), the permeability value (PSe) corresponding to the endothelium alone was then divided by the insert’s porous membrane surface area. To assess possible adsorption to plastics or non-specific binding to cells, the mass balance (%) was calculated from the amount of compound recovered in both compartments at the end of the experiment divided by the total amount added in the donor compartment at 0 min. Pe values were not calculated for compounds with a mass balance value below 90% and above 110%. Cellular accumulations of nanoparticles were normalized with the protein concentration measured using Bradford method (BioRad, Oxford, UK).

### 3.11. Statistical Analysis 

Descriptive statistics (*n*, means, SD, SEM) and statistical analyses were performed using the Prism 5.0 software (Prism Software, Irvine, CA, USA). The non-parametric Mann–Whitney Student’s *t*-test was used with confidence interval of 95%.

## 4. Conclusions

In this present research, we demonstrated that the extracts of the grape seed and grape skin strongly inhibit Aβ_(1–42)_ fibril formation by ThT binding assays and TEM ultrastructural analysis. The results indicated that the extracts have a more pronounced inhibitory effect in comparison with pure resveratrol. At the concentration of 40 μM, resveratrol inhibits around 86% of aggregation of Abeta peptide, the grape skin extract inhibits around 92%, and the grape seed extract inhibits around 97%. At the concentration of 80 μM, these values increased for 91%, 97% and 98%, respectively. These results suggest that the extracts beyond to contain resveratrol that inhibits the aggregation of Aβ_(1–42)_, they have other polyphenols that make this inhibitory effect most pronounced. 

Also, to overcome the rapid elimination of resveratrol from the blood, SLNs were engineered to encapsulate and transport the extracts into the brain where amyloid fibril formation occurs. The synthesized nanoparticles exhibit high encapsulation efficiency of the compounds studied. Overall, the SLNs are a promising dynamic system for the targeted delivery of grape extracts, as a natural substitute of resveratrol, to the brain in order to inhibit the formation of Aβ_(1–42)_ aggregates, thus potentially preventing or lowering the progression of AD.

These nanoparticles were functionalized with OX26 antibodies to target the BBB. Stability studies were performed to assess the use of these SLNs as a promising future drug delivery system, and the results showed that the nanoparticles are stable for a minimum period of one month. 

Then, we investigated the ability of SLNs functionalized or not to be trapped and transported by BBB cells using an in vitro model of the human BBB. This model consists to cultivate endothelial cells derived from hematopoietic umbilical cord blood cells seeded on a transwell^®^ filter, with brain pericytes. This model shows a low paracellular passage of low molecular weight molecules and presence of receptors and transporters expressed in vivo allowing its use for predict CNS distribution of compounds in human brain, transcytosis of NPs across the BBB and barrier’s physiology. Our results showed that the decoration of SLN with OX-26 antibody increases the SLN uptake by BBB cells and transcytosis across the BBB when compared with another antibody (LB 509) or without antibody (SLN alone). Even so, it is important to highlight that further studies are required to evaluate the potential of the OX-26 antibody coupled SLN as carriers for resveratrol and other drugs. Also, these results demonstrate that this kind of functionalization of SLN can be used for targeting the BBB and may be applied to transport other drugs to the brain.

## Figures and Tables

**Table 1:** Mean size of the nanoparticles with the grape skin and seed extracts encapsulated.

**Encapsulated Extract**	**Quantity of Extract (mg)**			
	2	5	10	15
	**Nanoparticle Diameter (nm)**			
**Grape skin**	187 ± 3	184 ± 8	182 ± 6	188 ± 18
**Grape seed**	168 ± 10	174 ± 12	188 ± 9	189 ± 2

**Table 2:** Entrapment efficiency of the grape skin and seed extracts in the nanoparticles.

**Encapsulated Extract**	**Quantity of Extract (mg)**			
	2	5	10	15
	**Entrapment Efficiency (%)**			
**Grape skin**	100 ± 20	100 ± 12	92 ± 7	75 ± 7
**Grape seed**	97 ± 2	86 ± 27	95 ± 2	97 ± 2

**Table 3:** Stability of the solid lipid nanoparticles encapsulated with resveratrol and grape seed and skin extract or unload nanoparticles at room temperature.

SLN	Size (nm)		Zeta Potential (mV)		Entrapment Efficiency (%)	
	0 day	2 months	0 day	2 months	0 day	2 months
Unloaded	142 ± 10	172 ± 3	−0.08	−0.21	-	-
Grape skin	182 ± 6	166 ± 10	−0.07	−0.02	92 ± 7	88 ± 10
Grape seed	188 ± 9	197 ± 20	0.34	−0.04	95 ± 2	97 ± 3

**Table 4:** Size, polydispersity index and zeta potential of the nanoparticles with and without conjugation of mAbs.

SLN	Size (nm)	Polydispersity Index	Zeta Potential (mV)
Without mab	233 ± 10	0.13 ± 0.03	−5.4 ± 0.5
With LB 509 mab	249 ± 1	0.14 ± 0.05	−5.0 ± 0.1
With OX26 mab	254 ± 17	0.23 ± 0.05	−4.0 ± 0.1

**Table 5:** Chemical structure of the lipids used in the solid lipid nanoparticles synthesis.

Name	Structure
Cetylpalmitate	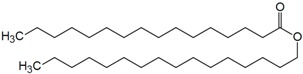
Polysorbate 80	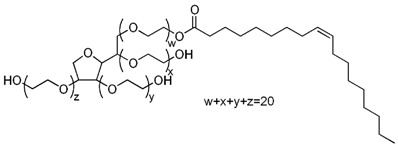
DSPE-PEG(2000) Maleimide: 1,2-distearoyl-*sn*-glycero-3-phosphoethanolamine-*N*-(maleimide(polyethyleneglycol)-2000)	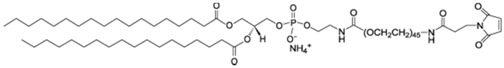
Liss Rhod PE: 1,2-dipalmitoyl-*sn*-glycero-3-phosphoethanolamine-*N*-(lissamine rhodamine B sulfonyl)